# Increasing the diversity of dietary fibers in a daily-consumed bread modifies gut microbiota and metabolic profile in subjects at cardiometabolic risk

**DOI:** 10.1080/19490976.2022.2044722

**Published:** 2022-03-20

**Authors:** Harimalala Ranaivo, Florence Thirion, Christel Béra-Maillet, Susie Guilly, Chantal Simon, Monique Sothier, Laurie Van Den Berghe, Nathalie Feugier-Favier, Stéphanie Lambert-Porcheron, Isabelle Dussous, Loïc Roger, Hugo Roume, Nathalie Galleron, Nicolas Pons, Emmanuelle Le Chatelier, Stanislav Dusko Ehrlich, Martine Laville, Joël Doré, Julie-Anne Nazare

**Affiliations:** a Univ-Lyon, CarMeN Laboratory, Inserm, Inrae, Université Claude Bernard Lyon-1, Oullins, France; b Centre de Recherche En Nutrition Humaine Rhône-Alpes, Univ-Lyon, CarMeN Laboratory, Université Claude Bernard Lyon1, Hospices Civils de Lyon, Cens, Fcrin/force Network, Pierre-Bénite, France; c Université Paris-Saclay, INRAE, MGP, Jouy-en-Josas, France; d Université Paris-Saclay, INRAE, AgroParisTech, Micalis Institute, Jouy-en-Josas, France; e Bridor, Rennes, France

**Keywords:** Dietary fiber diversity, cardiometabolic profile, gut microbiota, *Parabacteroides distasonis*, *Eisenbergiella sp*., glycoside hydrolases

## Abstract

Some cardiometabolic risk factors such as dyslipidemia and insulin resistance are known to be associated with low gut microbiota richness. A link between gut microbiota richness and the diversity of consumed dietary fibers (DF) has also been reported. We introduced a larger diversity of consumed DF by using a daily consumed bread in subjects at cardiometabolic risk and assessed the impacts on the composition and functions of gut microbiota as well as on cardiometabolic profile. Thirty-nine subjects at cardiometabolic risk were included in a double-blind, randomized, cross-over, twice 8-week study, and consumed daily 150 g of standard bread or enriched with a 7-dietary fiber mixture (5.55 g and 16.05 g of fibers, respectively). Before and after intervention, stool samples were collected for gut microbiota analysis from species determination down to gene-level abundance using shotgun metagenomics, and cardiometabolic profile was assessed. Multi-fiber bread consumption significantly decreased *Bacteroides vulgatus*, whereas it increased *Parabacteroides distasonis, Fusicatenibacter saccharivorans*, an unclassified Acutalibacteraceae and an unclassified *Eisenbergiella* (q < 0.1). The fraction of gut microbiota carrying the gene coding for five families/subfamilies of glycoside hydrolases (CAZymes) were also increased and negatively correlated with peaks and total/incremental area under curve (tAUC/iAUC) of postprandial glycemia and insulinemia. Compared to control bread, multi-fiber bread decreased total cholesterol (−0.42 mM; q < 0.01), LDL cholesterol (−0.36 mM; q < 0.01), insulin (−2.77 mIU/l; q < 0.05), and HOMA (−0.78; q < 0.05). In conclusion, increasing the diversity of DF in a daily consumed product modifies gut microbiota composition and function and could be a relevant nutritional tool to improve cardiometabolic profile.

## Introduction

Cardiometabolic diseases (CMD) are among the leading worldwide causes of death. Indeed, ischemic heart disease, stroke, and diabetes accounted for about 16.8 million deaths in 2019. Adiposity, dyslipidemia, and insulin resistance are known to increase the risk of developing CMD.^1^ Studies focusing on gut microbiota analysis have shown that such deleterious phenotypes are associated with low gut microbiota gene richness.^2^ Furthermore, studies comparing geographically distinct populations, both close and distant, have shown that an increased consumption of fruits and vegetables, thus higher quantity in dietary fiber intake, is associated with higher gut microbiota diversity.^3–7^ In overweight and obese subjects initially presenting a low gene count, a short-term energy-restricted diet including an increased quantity of dietary fibers, partly restored gut microbiota richness in parallel to improvement of the cardiometabolic profile.^8^ Taken together, this supports a key role of diet, particularly dietary fiber, in gut microbiota modulation and potentially in cardiometabolic risk factor management;^9^ especially since average dietary fiber intake is less than the recommended 25–30 g/day.^10,11^

Interestingly, a link between gut microbiota richness and consumed vegetable diversity has also been shown,^12^ suggesting that beyond quantity, the diversity of dietary fiber intake may also affect gut microbiota richness. Moreover, in a pig model, in the context of a high-fat diet, an improved control of lipid metabolism has been reported following supplementation with a mixture of three dietary fibers.^13^ Dietary fibers are mainly composed of resistant starch and non-starch polysaccharides (NSPs) from plant cell walls, particularly found in legumes, vegetables, and cereals. Other types of carbohydrates such as oligosaccharides, gums, and sugar-alcohols also reach the colon after escaping digestion in the small intestine. Fiber NSPs are complex polymers of cellulose, hemicellulose (xylan, xyloglucan, mannans, mixed-linkage glucans …) and pectins composed of a central sugar backbone, some branched with various side-chains by different glycosidic linkage patterns to form large, diverse molecules. All these polysaccharides are embedded in a complex matrix and represent various substrates for the gut microbiota, and particularly promote fibrolytic and glycolytic microbes that are genetically equipped with a panel of enzymes, substrate-binding proteins, and transporters to effectively hydrolyze these complex polymers.^14^ The human gut microbiota harbors 80 to 160 families of carbohydrate active enzymes (CAZymes) in healthy humans from different countries, compared to the 4 enzyme families of the human genome, which only break down starch, trehalose, and sugars in human milk.^15,16^ Thus, the more complex the structure of carbohydrates, the more the enzyme systems must be diversified to allow bacteria to completely break down complex fibers and convert sugar units into energy for its maintenance and multiple-host benefits.^17^ In turn, fiber breakdown delivers oligosaccharides that may feed the overall microbial community organized in a trophic network and promote a more global functional impact. The diversity and characteristics of fibers are thus thought to play a key role in this selective or global modulation of commensal bacteria and the host benefits.^18^

In this cross-over study, we thus aimed at increasing the quantity but also the diversity of dietary fiber intake using a daily consumed product such as sourdough bread and evaluated the induced effects on gut microbiota composition and function as well as on cardiometabolic risk profile. For this purpose, we defined a mixture of 7 dietary fibers of different molecular structures, with various physiochemical properties (both soluble and insoluble). We hypothesized that this particular mixture of dietary fibers would promote efficient fiber degradation by engaging a panel of enzymes with complementary modes of action. Due to the nature and metabolism of fibers, an impact on the cardiometabolic profile is also expected in parallel with the modification of the gut microbiota composition and functions.

## Results

### Participants characteristics

**Figure 1.**
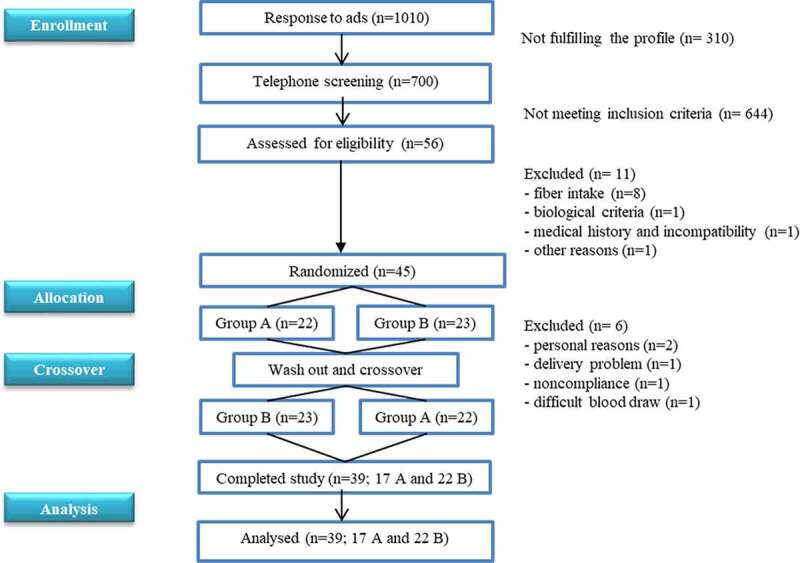
Consort flow diagram of participants.

Forty-five subjects with cardiometabolic risk profile were included and randomized: 39 completed the cross-over study (17 men, 22 women) and were analyzed (Figure 1). Ninety-two percent were dyslipidemic (high triglycerides (TG) and/or low high-density lipoprotein cholesterol (HDL-C) and/or high low-density lipoprotein cholesterol (LDL-C) and/or high total cholesterol (TC). They had neither fasting hyperglycemia nor diabetes nor elevated hs-CRP (high-sensitivity C-Reactive Protein) nor hypertension (Table 1).

**Table 1.** Subjects’ anthropometric, metabolic characteristics and habitual dietary fiber intake at baseline (n = 39)

	female			male		
	n = 22			n = 17		
Age (years)	43.0	±	10.6	41.6	±	12.9
Weight (kg)	75.7	±	8.9	93.9	±	10.8
Height (m)	1.6	±	0.1	1.8	±	0.1
BMI (kg/m^2^)	28.4	±	2.6	29.0	±	3.1
Waist circumference (cm)	97.1	±	6.6	103.9	±	7.9
Hip circumference (cm)	110.0	±	6.0	108.4	±	6.7
Blood biomarkers						
TG (mmol/L)	1.2	±	0.4	1.3	±	0.6
TC (mmol/L)	5.8	±	1.0	5.2	±	0.7
HDL-C (mM)	1.4	±	0.3	1.1	±	0.2
LDL-C (mM)	3.9	±	0.9	3.5	±	0.6
Glucose (mmol/L)	4.7	±	0.3	5.1	±	0.5
hs-CRP (mg/L)	3.0	±	3.1	3.7	±	5.6
HbA1c %	5.2	±	0.2	5.3	±	0.4
Blood pressure (mmHg)						
Systolic pressure	120.0	±	13.2	125.1	±	12.4
Diastolic pressure	71.9	±	11.0	78.9	±	9.2
Habitual dietary fiber intake						
Quantity (g/d)	17.45	±	1.6	16.3	±	2.3
Diversity (food group/d)	3	±	0.1	3	±	0.1

Data are expressed as mean ± SD. BMI: body mass index; TG: triglyceride; TC: total cholesterol, HDL-C: high-density lipoprotein cholesterol, LDL-C: low-density lipoprotein cholesterol; hs-CRP: high sensitivity C reactive protein; HbA1c: glycated hemoglobin.

### Participants dietary intake and compliance

The mean compliance to the dietary interventions (control (CTL) and multi-fiber (MF) bread consumption) was high (99%). Dietary records showed that subjects did not modify their energy and macronutrient intakes throughout the study. Likewise, the daily dietary fiber intake (without including consumed bread) in quantity and diversity remained low (<20 g/day, <3 fiber-rich food group/day) and was not modified throughout the study (Table S1). Consumed fiber-rich food items were mainly from starchy food, vegetables, and fruits (Figure S1).

### Impact of dietary interventions on gut microbiota composition

#### Only the multi-fiber bread significantly modified gut microbiota composition

**Figure 2.**
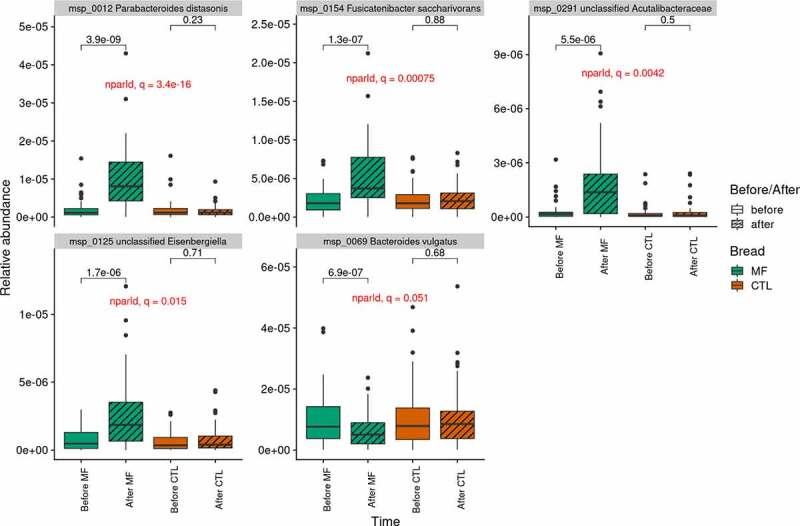
Abundance of the MSP significantly impacted by multi-fiber bread (n=39).

The two-month consumption of MF bread altered gut microbiota composition by modifying the relative abundance of specific gut bacterial species. *Bacteroides vulgatu*s msp_0069 significantly decreased (cliff’s delta (CD), effect size: −0.27, small), whereas *Parabacteroides distasonis* msp_0012 (CD: 0.72, large), *Fusicatenibacter saccharivorans* msp_0154 (CD: 0.49, large), the unclassified *Acutalibacteraceae* msp_0291 (CD: 0.47, medium), and the unclassified *Eisenbergiella* msp_0125 (CD: 0.46, medium) significantly increased (q ≤ 0.1) (Figure 2). CTL bread did not significantly alter gut microbiota composition. Consistently, when assessing intra-individual changes, of species relative abundance, based on Bray-Curtis dissimilarity index before and after each bread consumption, we observed that compared to control bread, Bray-Curtis dissimilarity index of gut microbiota was larger with multi-fiber bread consumption 0.38 ± 0.08 vs 0.34 ± 0.10 (p = .02) (Figure S2).

Increased MSP (panel 1-5); decreased MSP (panel 6). MSP: Metagenomic Species; MF: multi-fiber bread; CTL: control bread. P-values from Wilcoxon signed-rank test are displayed.

The washout period was appropriate. When assessing intra-individuals Bray-Curtis dissimilarity index between the different visits in the group that consumed MF bread and the group that consumed CTL bread during the period 1, we actually showed that there was no difference concerning the dissimilarity between V2 and V5 in the two groups (p = .75) although the dissimilarity between V2 – V4 was different (p = .06). Moreover, the species, which were increased or decreased in abundance with the multi-fiber bread showed no significant difference between V2 (baseline) and V5 (after wash-out) (Figure S3).

**Figure 3.**
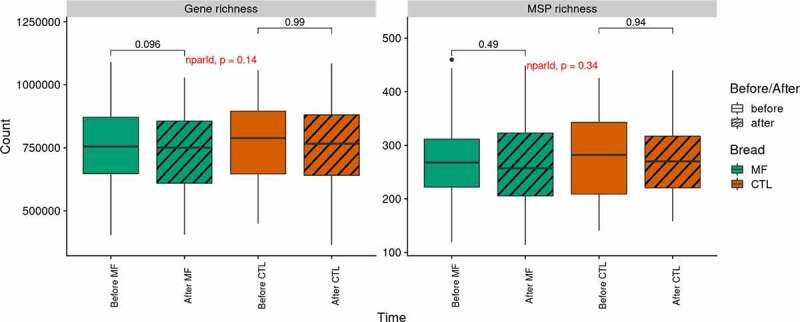
Gut microbiota richness throughout the interventions (n=39).

All along the dietary intervention, microbiota richness in terms of gene count and MSP (Metagenomic Species) count did not significantly differ (CD: 0.07 and 0.04 negligible respectively) (Figure 3).

Gene and MSP richness (left and right panel, respectively) evolution per bread. MSP: Metagenomic Species; MF: multi-fiber bread; CTL: control bread. P-values from Wilcoxon signed-rank test are displayed.

**Figure 4.**
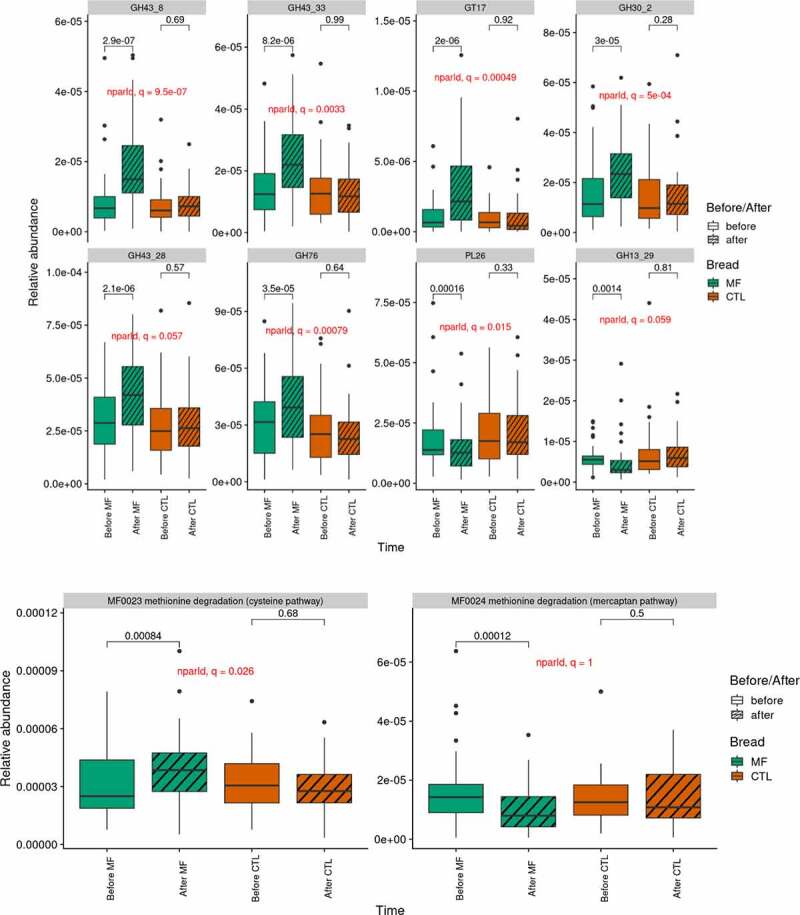
Gut microbiota function (n=39)

Gene abundance of CAZymes (A) and GMM (B) impacted by multi-fiber bread. GMM: gut metabolic modules; MF: multi-fiber bread; CTL: control bread. P-values from Wilcoxon signed-rank test are displayed. GH: glycoside hydrolase; GT: glycosyl transferase; PL: polysaccharide lyase.

### Impact of dietary interventions on gut microbiota functions

#### Only the multi-fiber bread significantly modified gut microbiota functions

The bioconversion potential of dietary fibers according to the microbial CAZymes patterns was assessed as the fraction of gut microbiota carrying a given CAZyme, i.e. the sum of species (MSP) with at least one gene annotated with this CAZyme (see Methods). Out of the 200 examined CAZyme families, MF bread impacted the relative abundance of CAZymes from eight different families and sub-families, among which six were significantly increased and two significantly decreased (absolute CD ranging from 0.23, small to 0.58, large, q < 0.1), while remaining stable with control bread (Figure 4). The increased CAZymes belong to glycoside hydrolase (GH) families (GH30_2, GH43_8, GH43_28, GH43_33, and GH76) and glycosyl-transferase (GT) family (GT17), while the decreased CAZymes belong to polysaccharide lyase (PL) family (PL26) and GH13_29 subfamily. Among the increased putative GHs, two GH30_2 proteins shared 51% and 52% aminoacid (aa) identity with a characterized β-D-xylosidase from rumen bacterium (ADO20355), respectively. One putative GH43_8 protein has 37% aa identity with a β-D-galactofuranosidase from *Bacteroides salyersiae* (EIY66405) and a GH43_33 sequence shared 39% aa identity with an α-L-arabinofuranosidase from *Halothermothrix orenii* H168 (ACL70803). Two GH76 putative proteins presented 31% and 38% aa identity with an endo-α-1,6-mannanase from *Bacillus circulans* (BAA75632), respectively. No characterized protein sequence was yet available for similarity search to the GH43_28 subfamily. Consistently, all the five increased putative GH families were found in *P. distasonis* msp_0012, two of them were also found in *F. saccharivorans* msp_0154 (GH43_28) and in the unclassified *Eisenbergiella* msp_0215 (GH76), respectively. Furthermore, the protein sequences of GH30_2, GH43_8, GH43_28, GH43_33, and GH76 were identical to those of several strains of *P. distasonis* already annotated for CAZymes (http://www.cazy.org/bP.html). Homologous proteins were also found in numerous *P. distasonis* sequences available in the databases (not shown).

Regarding gut-metabolic modules (GMM), only the cysteine pathway of methionine degradation (methionine = > L-homocysteine) changed significantly during the intervention and was increased with MF bread consumption (CD: 0.32, small; p = .026) (Figure 4). Interestingly, the mercaptan pathway of methionine degradation (methionine = > methanethiol) showed the opposite evolution with similar effect size, non-significantly after multiple test, though (p = .04, q = 1). Short-chain fatty acids-related GMM were not modulated during the intervention.

### Impact of dietary interventions on metabolic parameters

#### The multi-fiber bread significantly improve lipid profile and insulin sensitivity

**Figure 5.**
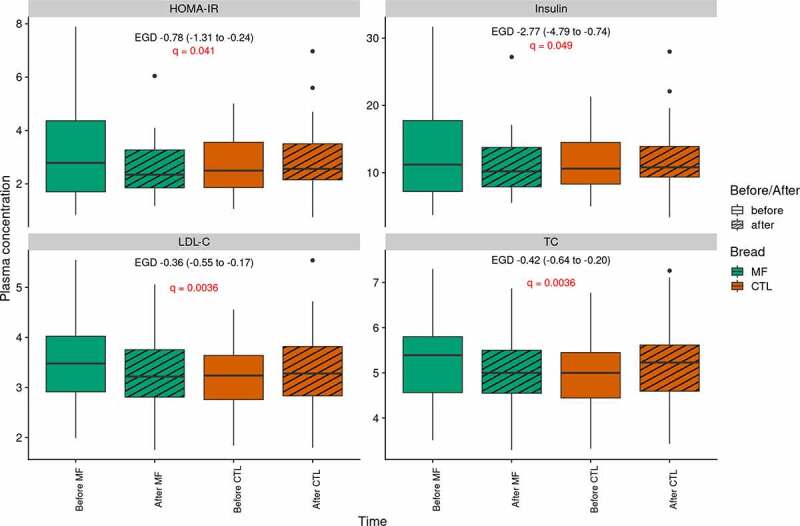
Effects of multi-fiber bread compared to control bread on lipid profile and insulin sensitivity (n=39).

Compared to CTL bread, MF bread significantly decreased total cholesterol (TC: −0.42 mM), low-density lipoprotein cholesterol (LDL-C:-0.36 mM), insulin (−2.77 mUI/l) and homeostasis model assessment (HOMA: −0.78) (q < 0.05) (Figure 5). There was no significant impact of MF bread on anthropometric and inflammatory parameters as well as on the postprandial metabolism of glucose in response to a standardized test meal challenge (Table 2).

Plasma concentrations of clinical variables impacted by multi-fiber bread. MF: multi-fiber bread; CTL: control bread. HOMA IR: homeostasic model assessment of insulin resistance; TC: total cholesterol; LDL-C: low-density lipoprotein cholesterol. EGD: estimated group difference. P-values from mixed linear model for repeated measures are displayed.

**Table 2.** Effects of multi-fiber bread compared to control bread on metabolic parameters (n = 39)

	Fiber-enriched bread						Control Bread						Effect of multi-fiber bread			
	Baseline			Post-Intervention			Baseline			Post-Intervention			EGD	95% IC		
*Anthropometric parameters*																
Weight (kg)	84.65	±	13.7	84.79	±	13.35	84.59	±	13.3	84.67	±	13.43	0.06	(−0.65	to	0.78)
BMI (kg/m^2^)	29.02	±	2.76	29.1	±	2.70	29.03	±	2.81	29.05	±	2.72	0.06	(−0.19	to	0.30)
Fat mass (kg)	32.86	±	8.11	32.7	±	8.04	32.83	±	8.19	32.83	±	8.06	0.11	(−0.48	to	0.69)
Waist circumference (cm)	100.23	±	8.37	100.28	±	8.44	99.74	±	8.24	100.17	±	8.02	−0.38	(−1.64	to	0.87)
Height circumference (cm)	109.91	±	5.87	110.05	±	5.99	109.79	±	5.88	110.28	±	6.21	−0.35	(−1.15	to	0.46)
*Fasting metabolic parameters*																
Glucose (mM)	5.35	±	0.49	5.22	±	0.40	5.31	±	0.48	5.29	±	0.40	−0.11	(−0.26	to	0.04)
NEFA (µM)	446.1	±	159.15	438.28	±	163.74	494.1	±	161.99	484.87	±	221.14	1.41	(−90.6	to	93.4)
Insulin (mIU/l)	13.41	±	7.63	10.94	±	4.37	11.34	±	4.18	11.96	±	4.89	−2.77*	(−4.79	to	−0.74)
HOMA	3.23	±	1.92	2.54	±	1.01	2.69	±	1.04	2.83	±	1.23	−0.78*	(−1.31	to	−0.24)
TG (mM)	1.32	±	0.51	1.26	±	0.43	1.29	±	0.59	1.28	±	0.49	−0.05	(−0.23	to	0.14)
Total cholesterol (mM)	5.29	±	0.92	5.03	±	0.86	4.97	±	0.78	5.13	±	0.85	−0.42**	(−0.64	to	−0.20)
HDL cholesterol (mM)	1.24	±	0.27	1.19	±	0.30	1.19	±	0.26	1.20	±	0.31	−0.05	(−0.11	to	0.00)
LDL cholesterol (mM)	3.46	±	0.80	3.27	±	0.72	3.20	±	0.66	3.36	±	0.77	−0.36**	(−0.55	to	−0.17)
CRPus (mg/L)	3.36	±	6.47	3.41	±	4.68	4.00	±	5.69	2.93	±	4.28	0.28	(−0.22	to	0.79)
RMR (kcal)	1490.27	±	330.33	1699.97	±	332.94	1717.04	±	359.58	1700.98	±	334	0.01	(−0.02	to	0.05)
CD14 (µg/mL)	1.53	±	0.42	1.45	±	0.42	1.57	±	0.42	1.48	±	0.32	0.01	(−0.19	to	0.21)
LBP (µg/mL)	16.06	±	6.94	16.33	±	5.80	16.20	±	6.51	15.81	±	7.49	0.67	(−2.27	to	3.61)
Ratio LBP/CD14	10.81	±	4.69	12.69	±	8.75	10.55	±	4.38	10.96	±	5.3	1.67	(−1.26	to	4.61)
Postprandial metabolic parameters																
Glucose tAUC (mM*min)	1031.3	±	93.8	1024.47	±	73.46	1048.32	±	96.24	1043.61	±	122.98	−2.12	(−40.78	to	36.54)
Glucose iAUC (mM*min)	147.16	±	118.39	151.14	±	103.46	197.89	±	132.42	192.77	±	151.53	9.10	(−41.02	to	59.23)
Glucose peak (mM)	7.26	±	1.04	6.99	±	0.76	7.25	±	1.09	7.18	±	1.05	−0.2	(−0.62	to	0.23)
Insulin tAUC (mUI/l*min)	7676	±	2951.96	7568.86	±	3181.22	7707.86	±	3098.25	7694.25	±	3568.1	24.18	(−1090	to	1139)
Insulin iAUC (mUI/l*min)	5827.22	±	2804.61	5837.54	±	2941.38	5991.59	±	2901.58	5996.27	±	3252.8	112	(−1085	to	1308)
Insulin peak (mIU/l)	90.28	±	51.08	87.39	±	63.01	86.27	±	41.12	88.26	±	57.99	−6.44	(−28.88	to	15.99)

Data are expressed as mean ± SD. Effects of multi-fiber bread were analyzed using linear mixed model for repeated measures with heterogeneous Toeplitz or autoregressive as covariance structure. MF: multi-fiber bread; CTL: control bread; BMI: body mass index; RMR: resting metabolic rate; HOMA IR: homeostasic model assessment of insulin resistance; TC: total cholesterol; HDL cholesterol: high-density lipoprotein cholesterol; LDL-C: low-density lipoprotein cholesterol, TG: triglyceride; NEFA: non-esterified fatty acid; hs-CRP: high sensitivity C-reactive protein. EGD: estimated group difference. Adjusted p value *< 0.05 ** <0.01.

### Association between gut microbiota composition and function, and metabolic parameters

**Figure 6.**
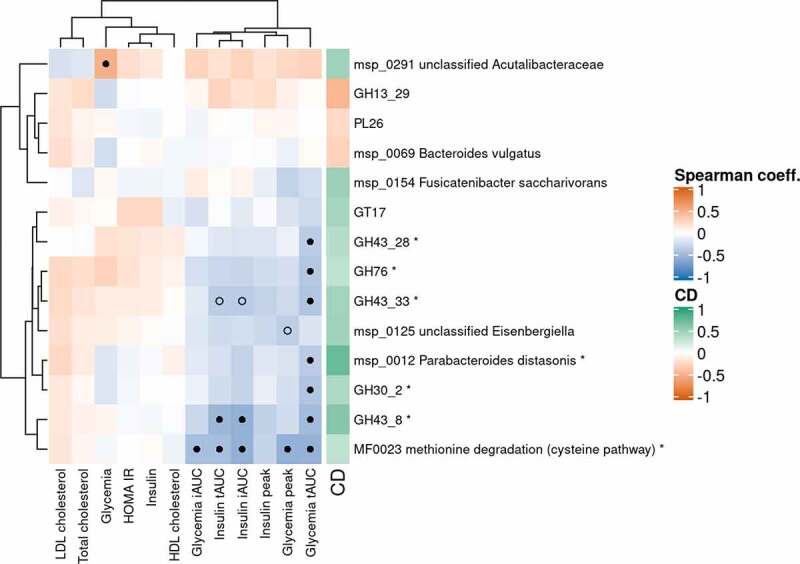
Correlation between metagenomic features and clinical variables (n=39).

We correlated the delta (after – before multi-fiber bread consumption) of impacted metagenomics features with those of clinical variables related to cholesterol and glucose metabolisms (Figure 6). We showed that a group of features enriched after multi-fiber bread consumption, *P. distasonis*, an unclassified *Eisenbergiella*, methionine degradation, and a set of GHs (GH30_2, GH43_8, GH43_28, GH43_33, GH76) were negatively correlated with the peaks and total/incremental area under curve (tAUC/iAUC) of postprandial glycemia and insulin, in response to a standardized test meal challenge.

Heatmap of Spearman’s correlation coefficient between deltas (after – before multi-fiber bread) of metagenomics features impacted by MF bread (in rows) and deltas of clinical variables related to cholesterol and glucose metabolisms (in columns). The right side bar stands for the metagenomics features Cliff’s Delta (red: decreased with MF, green: increased with MF). Black dots stand for correlations that are significant after Benjamini-Hochberg correction (q ≤ 0.1). Empty circles stand for correlations that are significant regarding non-corrected p-values (p ≤ 0.05). *P. distasonis* and the modules/CAZymes it carries are denoted with ‘*’ at the end of their name. CD: Cliff’s Delta; GH: glycoside hydrolase; GT: glycosyl-transferase; PL: polysaccharide lyase.

## Discussion

To our knowledge, this study investigated for the first time the impact of increasing the diversity of the dietary fiber intake, beyond the quantity, on the composition and the functions of the gut microbiota as well as on cardiometabolic profiles using a daily consumed product such as bread. We demonstrated that in subjects at cardiometabolic risk, such interventions alter gut microbiota composition and function by modifying the relative abundance of specific gut bacterial species, CAZymes and GMM, accompanied by a significant improvement of cholesterol and insulin sensitivity parameters.

In this study, we showed that in a population of subjects at cardiometabolic risk of whom 92% were dyslipidemic, 2 months of consumption of multi-fiber bread significantly decreased total cholesterol (−0.42 mM) and LDL cholesterol (−0.36 mM) compared to 2 months of consumption of standard bread. Epidemiological studies have shown an inverse association between dietary fiber intake and cholesterol levels.^19^ Dietary intervention trials (2 to 16 weeks), based on different fiber-enriched diets with a dose varying from 5 to 23 g/d and conducted in dyslipidemic subjects, consistently showed a significant mean decrease of total and LDL cholesterol between 0.1 and 0.3 mM and 0.1 to 0.2 mM, respectively.^20–22^ Such decrease of LDL cholesterol is important in terms of reducing cardiometabolic risks, as 1 mM LDL cholesterol reduction was shown to be associated with a 19% lower risk of coronary mortality^23^ and a much greater decrease of 52% CM risk in case of early intervention.^24^

Literature suggests that the cholesterol-lowering effect of dietary fiber is due to different factors. First, the transformation of dietary cholesterol to coprostanol by coprostanoligenic bacteria including *Bacteroides* sp. D8 closely related to *B. vulgatus*, based on its morphological and biochemical characteristics.^25^ However, we reported here a decrease of *B. vulgatus* not supporting a major impact of this mechanism on the cholesterol-lowering effect of the mixture of fibers. Then, fibers are also known to affect bile acids. In the colon, bacteria can modify bile acids by hydrolyzing them, resulting in cholesterol-lowering. Bacteria capable of such reactions are those with bile salt hydrolase activity including bacteria belonging to *Bacteroides, Parabacteroides* and *Clostridium* genera^26,27^ and particularly *P.distasonis*.^28,29^ Since *P.distasonis* was significantly increased after multi-fiber bread, this mechanism could have been involved in the cholesterol-lowering effect of the mixture of fibers.

Interestingly, multi-fiber bread consumption significantly improved insulin sensitivity parameters. Epidemiological studies have reported a positive relation between dietary fiber intake and insulin sensitivity.^30,31^ A clinical study conducted in overweight and at cardiometabolic risk subjects has reported an improvement of insulin sensitivity estimated by HOMA following diet incorporating resistant starch, with an average HOMA decrease of 10.4%.^32^ Other studies all involving at cardiometabolic risk subjects, supported that wholegrain, oat-based products, β-glucan extract, and refined-grain products had no impact on insulin sensitivity.^24–26^ Here, we demonstrated a much larger 21%-decrease of HOMA and thus insulin resistance.

In this study, we hypothesized that using the dietary fiber mixture instead of a single one would promote gut microbiota diversity and functions since it would stimulate the metabolism of a larger number of ecological niches and generate a larger variety of by-products usable by the entire microbial ecosystem. However, we did not detect any change in gut microbiota richness in our study. Previously, Cotillard et al. showed that in overweight and obese subjects, a short-term energy-restricted diet including an increased quantity of dietary fibers partly restored the gut microbiota richness, but only in the subgroup initially presenting a markedly lower gene count (LGC) (compared to a higher gene count subgroup (HGC). Due to differing sequencing technologies used in this study and that of Cotillard et al., it was not possible to robustly compare gut microbiota richness between the subjects from the two studies. However, it has been reported that the body mass index (BMI) negatively correlates with gut microbiota richness.^33,34^ As the subjects included in this study presented a lower BMI compared to those from Cotillard et al. (28.9 ± 2.8 vs 33.2 ± 0.6), their gut microbiota richness may be higher, which could explain why we did not impact this parameter. Consistently, when comparing our cohort to a nationality- and sequencing technology-matched overweight cohort^34^ (our cohort < BMI: 31.4 ± 3.0 < Cotillard et al. cohort), richness was 17% higher than for this cohort (data not shown). Baseline microbiota richness could therefore be an important inclusion criterion to consider for such studies. Although we managed to increase both the quantity and the diversity of dietary fiber intake of included subjects, modifying one component of the diet without energy restriction or change in macronutrient composition may not have been sufficient to significantly alter overall microbiota richness in this population. However, we observed a significant decrease in abundance of *B. vulgatus* and a significant increase of *P. distasonis, F. saccharivorans*, an unclassified Acutalibacteriaceae, and an unclassified *Eisenbergiella* after 2 months of multi-fiber bread consumption. Multi-fiber bread nutritional interventions thus more specifically targeted certain taxa. Previous studies have also evidenced an increase in the level of *P. distasonis* after a dietary intervention using either single fiber supplementation^35,36^ or mixtures.^37^

Most if not all, glycoside hydrolases families increased with multi-fiber bread consumption have been found in *P. distasonis*, emphasizing the role this species may play in fiber supplementation. Among them, GH43_8/28/33 and GH30_2 are thought to be debranching enzymes aiding in the degradation of NSPs such as arabinoxylan from hemicelluloses, and galactomannan found in gums and pectin, although very few information is available in the literature regarding these subfamilies (from zero to two characterized members). Furthermore, GH43 are among the most abundant CAZymes found in the human gut microbiome and probably access to a wide range of complex carbohydrates.^38^ All together, these enzymes families are consistent with the nature of plant cell wall polysaccharides or gums provided by the cereals of multi-fiber bread and locust bean gum, respectively. GH76 are putative α-1,6-endomannanases dedicated to the degradation of mannans from yeast glycoproteins. Both multi-fiber and control breads are sourdough bread, but the presence of various types of fibers in the multi-fiber bread during the fermentation process with yeasts appeared to enhance bacteria bearing enzymes that hydrolyze yeast cell wall mannans. By regularly consuming yeast-leavened bread and other fermented products, Bacteroidetes in the gut microbiota have evolved the capacity to metabolize these glycans, in particular *Bacteroides* and *Parabacteroide*s.^39^ Multi-fiber bread could therefore have enhanced this metabolic function through the increase of *P. distasonis*. Concerning the decreased families, GH13_29 includes α-glucosidase and α-phosphotrehalases related to the hydrolysis of starch and trehalose. Enzymes encoding-genes from this subfamily are rather observed in the genome of Enterobacteriacea, and lactic acid bacteria^40,41^ but many other GH13 enzymes dedicated to the disruption of alpha glycoside linkages are found in *P. distasonis* according to the CAZy database, PL26 is thought to degrade rhamnogalacturonan from pectins but only one fungal enzyme from this family has been characterized so far,^42^ therefore other functional characteristics may exist. Nevertheless, numerous PL26 encoding genes have been found in *Bacteroides* species including *B. vulgatus*^43^ which is also significantly decreased with MF bread consumption. *B. vulgatus* has been reported to be associated with alteration of insulin resistance.^44^ Thus, its observed decrease could partly explain the improvement of insulin sensitivity. Moreover, we showed a significant increase of *Parabacteroides distasonis* whose involvement in type 2 bile acid production and farnesoid X receptor (FXR) pathway activation resulting in insulin sensitivity improvement has recently been shown.^45^ Moreover, the methionine degradation to L-homocysteine was the metagenomics features that displayed the highest number of significant correlations with the postprandial glycemia and insulinemia parameters. Both methionine and homocysteine have been related to insulin resistance.^46–51^ Hence, the modulation of methionine metabolism through the increase of *P. distasonis* could be a possible mechanism to explain the decrease of insulin observed in this study.

It is well known that studying the impact of dietary interventions on human microbiota has some limitations, such as a limited number of participants like in our study, and the fact that studies normally provide fecal samples reflecting the microbiota of the distal colon, but do not allow access to the microbiota of the actual site of food fermentation (cecum and proximal colon).^52^ Beyond the effects on gut microbiota functions mediated by the modulation of the microbiome, we would expect observing effects on the metabolome.

In conclusion, increasing the diversity of dietary fibers in a daily consumed product modified gut microbiota composition and promoted GH families involved in the degradation of plant polysaccharides and locust bean gum found in the seven selected dietary fibers. The parallel improvement of lipid and insulin sensitivity parameters suggests that such intervention could be a relevant nutritional approach to improve cardiometabolic profile and further prevent cardiometabolic risk.

## Materials and methods

### Study participants

Forty-five subjects with CMR profile were included in this study between November 2017 and July 2018. Inclusion criteria included age: 18–70 years old, body mass index (BMI): 25–35 kg/m^2^, waist circumference >80 cm for women and >96 cm for men, daily bread consumption <200 g/day, low dietary fiber intake <20 g/day, stable weight and moderate physical activity, no known gastrointestinal disease, no previous bariatric surgery, no use of antibiotics or other drugs interfering with microbiota composition in the 3 months prior and during the study.

### Study design

This study was a single-center, randomized, double-blind crossover trial conducted at the Human Nutrition Research Center of Rhône-Alpes (CRNH-RA) and carried out in accordance with the Second Declaration of Helsinki and French Jardé’s law. It was reported and registered on http://www.clinicaltrials.gov (NCT03875898). All participants received and signed informed consent approved by the Scientific Ethics Committee of Bordeaux Sud-Ouest and Outre-Mer III.

After a 1-week run-in period (150 g/day of standard sourdough bread (control CTL)), subjects received two 8-weeks dietary interventions in a random order according to randomization. The two dietary interventions were: 1) 150 g/d of sourdough bread enriched with a mixture of 7 selected dietary fibers (multi-fiber; MF) and 2) 150 g/d of CTL. Group A started with MF, whereas group B started with CTL. A 4 to 6-weeks washout period and another run-in separated the two interventions (Figure S4). CTL and MF bread were structurally matched (color, size, and texture), although had different dietary fiber content (Table S2). 150 g of MF bread contained 16.05 g of 7 selected fibers with a 1:1 ratio of insoluble to soluble fiber.

Every week, baked and frozen breads were directly delivered to the subjects in individual sealed pack of 3 × 50 g and stored in their freezer until consumption. Compliance was checked by the number of returned empty packs.

During the intervention periods, subjects were asked to maintain the same diet in terms of caloric and macronutrients content and were ordered to fill in a 3-day dietary record. Data from this 3-day dietary record were processed using NUTRILOG© which allowed to estimate energy and macronutrient intake. In parallel, the subjects also completed a questionnaire concerning 128 fiber-rich food items to evaluate their dietary fiber intake in terms of diversity.^12^ A fiber-rich food group (breakfast and biscuits, starchy food, dried vegetables, vegetables, fruits, nuts, chocolate, and probiotics) is made of different items (for example, apple is an item belonging to fruits). For each subject, fiber-rich food group was taken into account when he/she ate at least one item/day of this group on 2 days out of three.

At the beginning and the end of each nutritional intervention period, metabolic assessment days were scheduled to evaluate CM risk factors (V2, V4, V5, and V7).

During the 3 days before metabolic assessment days, subjects were ordered to collect a stool sample at ambient temperature using a specific kit containing RNAlater® as stabilizing solution, following the International Human Microbiome Standards (IHMS, SOP 05) (http://www.human-microbiome.org/index.php?id=Sop&num=005) and send it to the French National Research Institute of Agriculture, food and Environment (INRAE) MetaGénoPolis (mgps.eu) for analysis. Intermediate visits with dietician were also scheduled to ensure a good compliance (V3 and V6).

On metabolic assessment days, subjects arrived at CRNH-RA after an 8 h overnight fast following the ingestion of a standard low dietary fiber evening meal (one serving of lean meat or fish, rice, a dairy product and fruit compote). Body weight, fat mass percentage, height, and waist circumference were measured using standardized methodologies with a calibrated weighing scale, a Bodystat Quadscan 4000 (BQ4000; Bodystat Ltd. Douglas, UK) stadiometer and non-elastic tape, respectively. BMI was calculated as weight/height^2^. The resting metabolic rate (RMR) was measured by indirect calorimetry using a QUARK calorimeter (Cosmed, Rome, Italy). Subjects were served a breakfast at T0 (the study product (multi-fiber or control bread), hot drink, jam, and butter) and a standardized challenge test meal at T240. Fasting and postprandial blood (T0, T15 T30 T45 T60 T90 T120 T180 T210 T240 T255 T270 T300 T330 T360 T390 T420) were collected using an antecubital vein catheter.

### Biochemical blood analyses

Collected blood was centrifuged immediately for 10 min at 4,500 rpm. Plasma was stored at −20°C until the assays were conducted.

Glycemia was measured by spectrophotometry according to Architect Abbott Hexokinase method; C-reactive protein (CRP) by immunoturbidimetry; Non-esterified Fatty Acid (NEFA) by colorimetric technic; total cholesterol (TC), HDL-cholesterol (HDL-C) and triglyceride (TG) by spectrometry using Architect Module Chimie Abbott method; insulin by radio immunoassay (RIA) according to RIA CisBio IBA method, LBP and sCD14 by sandwich ELISA according to CliniSciences instructions. LDL-cholesterol was calculated using Friedwald formula and HOMA as plasma glucose (mmol/L) × plasma insulin (mUI/L)/22.5.

### Microbiota analysis

#### DNA extraction of stool samples and shotgun sequencing

DNA extraction from aliquots of fecal samples was performed following the IHMS SOP 07 V2 (http://www.human-microbiome.org/index.php?id=Sop&num=005). DNA was quantitated using Qubit Fluorometric Quantitation (ThermoFisher Scientific, Waltham, US) and qualified using DNA size profiling on a Fragment Analyzer (Agilent Technologies, Santa Clara, US). Three µg of high molecular weight DNA (>10 kbp) was used to build the library. Shearing of DNA into fragments of approximately 150 bp was performed using an ultrasonicator (Covaris, Woburn, US) and DNA fragment library construction was performed using the Ion Plus Fragment Library and Ion Xpress Barcode Adapters Kits (ThermoFisher Scientific, Waltham, US). Purified and amplified DNA fragment libraries were sequenced using the Ion Proton Sequencer (ThermoFisher Scientific, Waltham, US), with a minimum of 20 million high-quality reads of 150 bp (in average) generated per library.

#### Microbial gene count table

To create the gene count table, the METEOR software was used: first, reads were filtered for low-quality by AlienTrimmer (https://forgemia.inra.fr/metagenopolis/meteor).^8^ Reads that aligned to the human genome (identity > 95%) were also discarded. Remaining reads were trimmed to 80 bases and mapped to the Integrated Gut Catalog 2 (IGC2),^53,54^ comprising 10.4 million of genes, using Bowtie2.^55^ First, the unique mapped reads (reads mapped to a unique gene in the catalog) were attributed to their corresponding genes. Second, the shared reads (reads that mapped with the same alignment score to multiple genes in the catalog) were attributed according to the ratio of their unique mapping counts of the captured genes. The resulting count table was further processed using the R package *MetaOMineR* v1.31.^2^ It was downsized to 14 million mapped reads to take into account differences in sequencing depth and in mapping rate across samples. Then the downsized matrix was normalized for gene length and transformed into a frequency matrix (fragments per kilobase of exon model per million reads mapped) (FPKM) normalization). Gene count was computed as the number of genes detected (i.e., whose abundance is strictly positive) in a given sample after downsizing.

#### Metagenomic Species (MSP) profiles

The IGC2 catalog was organized into 1990 Metagenomic Species (MSP), clusters of minimum 100 genes, using MSPminer^54^ MSP taxonomy was assigned with the Genome Taxonomy Database.^56^ Relative abundance of an MSP was computed as the mean abundance of its 100 ‘marker’ genes (that is, the genes that correlate the most altogether). If less than 10% of ‘marker’ genes were seen in a sample, the abundance of the MSP was set to 0. Relative abundances at higher taxonomical ranks were computed as the sum of the MSP that belong to a given taxa. MSP count was assessed as the number of MSP present in a sample (that is, whose abundance is strictly positive).

#### Microbiome functional potential

Genes from the IGC2 catalog were mapped with diamond^57^ onto KEGG orthologs (KO) from the KEGG database (version 8.9).^58^ Each gene was assigned to the best-ranked KO among hits with e-value <10–5 and a bit score >60. For a given sample, we assessed the abundance of a KEGG module or a Gut-Metabolic Modules (GMMs)^59^ with the following procedure:

- We restricted the gene content of each MSP detected in the sample to its genes also detected in the sample, plus the core genes of the MSP;
- We computed the fraction of the module present in the restricted set of genes for the pair sample/MSP;
- We considered that an MSP carried a module in a given sample if the computed fraction was above 90%;
- We summed the abundance of all MSPs that carried the module for a given sample.

The IGC2 was also annotated on the Carbohydrate-Active enZYmes Database (CAZy, November 2018) (http://www.cazy.org/^60^).^54^ The abundance of each CAZYme group (glycoside hydrolases (GH), glycosyl-transferases (GT), polysaccharide lyases (PL), and carbohydrate esterases (CE)) was computed using the same process as KEGG modules and GMM.

The abundance of each CAZyme group (glycoside hydrolases (GH), glycosyl-transferases (GT), polysaccharide lyases (PL), and carbohydrate esterases (CE)) was computed using the same process as KEGG modules and GMM.

### Statistics

The primary outcome of this study was an increase of 20% of gut microbiota richness which corresponds to 76 000 genes according to the distribution of gut microbiota richness of two cohorts of obese and overweight subjects.^2,8^ An increase of 76 000 genes allowed the individuals to move from Low Gene Count (LGC) status to High Gene Count (HGC) status. LCG and HGC individuals significantly differed on metabolic profile, HGC individuals presenting a better metabolic profile. Assessing the impacts on cardiometabolic profile was actually the secondary outcome of this study.

According to these data, to achieve a significance of p < .05 and a power (type II error) of 95%, a size of 40 subjects was required.

All statistical analyses and graphs were performed with R software v3.6.0 and SAS software 9.4 TS Level 1M6.^61^

To assess if a given metagenomic feature was impacted by the multi-fiber bread, we used the R package *nparLD*^62^ to perform non-parametric analysis of the longitudinal data. P-values from the interaction of the factors “bread” and “visit” were corrected for multiple test with the Benjamini–Hochberg procedure. A feature was considered significant when corrected p-values ≤0.1. Finally, Wilcoxon signed-rank test were performed on each bread separately (i.e., before/after multi-fiber bread, and before/after control bread) to assess which bread impacted the feature. Effect size was computed using the Cliff’s Delta with the R package *effsiz*e.^63^ This measure gives an information similar to log-fold change, but is comprised between −1 and 1. (0: no effect; +1 or −1: large effect). The magnitude of the effect size d is assessed as negligible if |d| <0.147, small if |d| <0.33, medium if |d| <0.474, and large otherwise.^64^

For clinical variables, the effect of multi-fiber bread was evaluated by calculating the estimated group difference, i.e., the difference between the changes induced by each of the two breads. A linear mixed model for repeated measures, with Toeplitz or autoregressive structure (AR) as covariance structure, was used to determine whether the difference between the changes induced by each of the two breads was statistically significant. Bread, time, period, and sequence were included as fixed variables. In order to account for variability between subjects and to adjust for any nonspecific differences, subjects were included as random effects. When the normality of the model residuals was not assumed, a logarithmic transformation of the data has been done.

Correlations between variables were performed using Spearman’s correlations. All p-values were adjusted for multiple testing with the Benjamini Hochberg Procedure. Unless stated otherwise, a corrected p-value is considered significant if inferior to 0.1; a non-adjusted p-value is significant if inferior to 0.05.

## Data Availability

The raw sequencing data are deposited into the European Nucleotide Archive (ENA) of EBI (https://www.ebi.ac.uk/ena/browser/home) under PRJEB48663. The study protocol and the datasets generated during and/or analyzed during the current study, including deidentified participant data will be available from the corresponding author on reasonable request.
